# Supplementary material for: Dual CXCR4 and E-Selectin Inhibitor, GMI-1359, Shows Anti-Bone Metastatic Effects and Synergizes with Docetaxel in Prostate Cancer Cell Intraosseous Growth
Source: Cells. 2019 Dec 20;9(1):32. doi: 10.3390/cells9010032 (PMC7017374; doi:10.3390/cells9010032)
Supplement: Supplementary file 1 [file cells-09-00032-s001.pdf]

A Statistical analysis performed on tumor weights shown in figure 4B

Treatment	Tumor weight (gr ± SE)	statistics
Controls (CTRL)	1,069 ± 0,220	
GMI-1359	0,725 ± 0,071	P=0,0026 vs CTRL
GMI-1271	0,925 ± 0,098	P=0,0643 vs CTRL<br>P=0,0005 vs GMI-1359
CTCE-9908	0,845 ± 0,090	P=0,0269 vs CTRL<br>P=0.0037 vs GMI-1359<br>P= 0,1247 (NS) vs GMI-1271
DTX	0,515 ± 0,129	P=0.0001 vs CTRL<br>P= 0,0021 vs GMI-1359<br>P=0,0004 vs CTCE-9908<br>P<0,0001 vs GMI1271
GMI-1359 + DTX	0,341 ± 0,079	P<0.0001 vs CTRL<br>P=0,0001 vs DTX<br>P=0,0026 vs GMI1359
CTCE-9908 + DTX	0,485 ± 0,035	P=0.0001 vs CTRL<br>P= 0,1854 (NS) vs DTX<br>P=0,0001 vs CTCE-9908<br>P=0,0187 vs GMI-1359 + DTX
GMI-1271 + DTX	0,510 ± 0,049	P=0.0007 vs CTRL<br>P=0,1815 (NS) vs DTX<br>P=0,0004 vs GMI-1271<br>P=0,0023 vs GMI-1359 + DTX<br>P=0,1199 (NS) vs CTCE-9908 + DTX

B Statistical analysis performed on TTPs shown in figure 4C

Treatment	TTP (days ± SE)	statistics
Controls (CTRL)	9,0 ± 1,9	
GMI-1359	13,6 ± 2,2	P=0,0120 vs CTRL
GMI-1271	10,4 ± 0,5	P=0,0997 (NS) vs CTRL<br>P=0,0013 vs GMI-1359
CTCE-9908	12,2 ± 2,2	P=0,0169 vs CTRL<br>P=0.8773 (NS) vs GMI-1359<br>P= 0,0355 vs GMI-1271
DTX	16,3 ± 1,1	P=0.0002 vs CTRL<br>P= 0,0013 vs GMI-1359<br>P=0,0225 vs CTCE-9908<br>P=0,0011 vs GMI1271
GMI-1359 + DTX	21,9 ± 1,8	P<0.0001 vs CTRL<br>P=0,0173 vs DTX<br>P=0,0029 vs GMI1359
CTCE-9908 + DTX	18,4 ± 0,4	P=0.0001 vs CTRL<br>P= 0,1854 (NS) vs DTX<br>P=0,0499 vs CTCE-9908<br>P=0,0377 vs GMI-1359 + DTX
GMI-1271 + DTX	16,7 ± 1,5	P<0.0001 vs CTRL<br>P=0,7655 (NS) vs DTX<br>P=0,0021 vs GMI-1271<br>P=0,0064 vs GMI-1359 + DTX<br>P=0,2446 (NS) vs CTCE-9908 + DTX

C Statistical analysis performed on Kaplan Meier shown in figure 5D-F

Comparison+ DTX	Hazard ratio	statistics
CTRL vs DTX	12.6	P<0.0001
CTRL vs GMI1359 + DTX	22,9	P<0.0001
DTX vs GMI139 + DTX	8,7	P=0.003
GMI 1359 vs GMI1359 + DTX	7,4	P=0,0004
CTRL vs CTCE9908 + DTX	16.2	P<0.0001
DTX vs CTCE9908 + DTX	14,8	P<0.0001
CTCE-9908 vs CTCE9908 + DTX	5,2	P=0.0063
CTCE9908 + RT vs GMI1359 + DTX	2,8	P=0.4476 (NS)
CTRL vs GMI1271 + DTX	14,2	P<0.0001
DTX vs GMI1271 + DTX	1,5	P=0.8756 (NS)
GMI1271 + DTX vs GMI1359 + DTX	7,9	P=0.0022
GMI1271 + DTX vs CTCE9908 + DTX	4,8	P=0.0377

Figure S1. Statistical analyses on graphs shown in Figures 4B,C and 5D–F. (A) Analysis on tumor weight data; (B) analysis on TTP data and (C) analyses of Kaplan Meier curves generated in Figures 5 D-F.

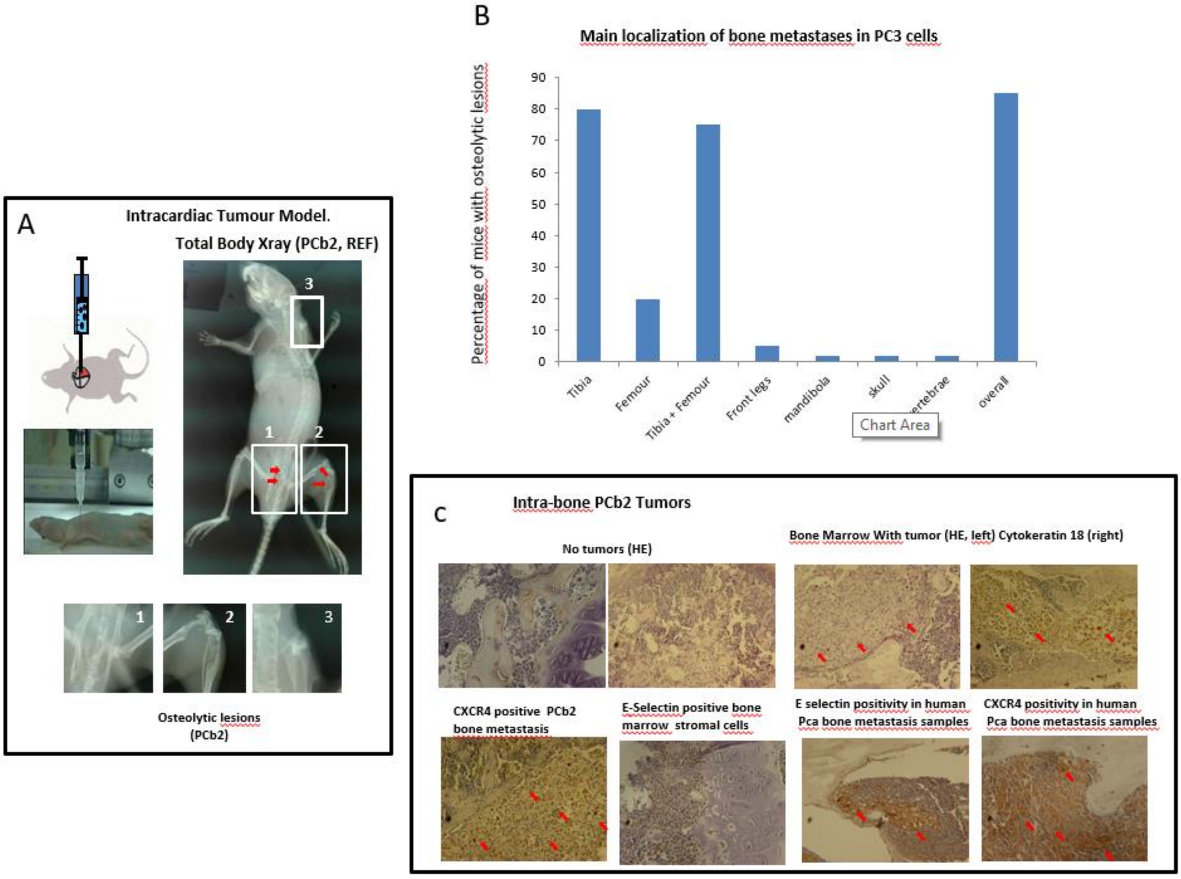

Figure S2. Intra-ventricular model. (A) modality for intraventricular cell injection and identification of three common metastatic osteolytic sites by Xray (see red arrows): (1) vertebral localization; (2) tibia and femur and (3) gleno-humeral joint. (B) percentage of bone engraftment (colonization) after intraventricular cell injection by using the osteolytic PCb2 model (C) histological intra-osseous localization of PCb2 cells indicating the presence of cytokeratin CK18 positive PCa cells mixed with bone marrow cells. CXCR4 stained cytosol and membranes from CXCR4 expressing cells. CXCR4 was evident in the cytosol of PCb2 cells selectin ligands was expressed in the cytosol of bone marrow and tumor cells (adjacent to bone marrow or trabecular bone). The immunohistochemical evaluation of CXCR4 and Heca-452 IR was performed also on human tissues derived from tissue microarray from Biomax as indicated in the Material and Methods section. The bar at the bottom of figure is 0.5 cm and represents 500 µm (5×), 250 µm (10×) and 125 µm (20×). Figure S3. CXCR4 modulates osteoblast proliferation: (A) osteoblasts were grown in presence of conditioned media from PC3, 22rv1, LnCaP, VCaP and C42B and stained with Crystal Violet that was solubilised and assayed for optical density (OD) measured at 595 nm. (B) Effects of GMI-1359, GMI-1271 and CTCE-9908 on C4-2B, PC3 and 22rv1 administration. (C) solubilization of Alazarin Red staining and analyses of densitometric values evaluated at 450 nm. Statistics: *$p < 0.05$
